# En bloc resection for primary spinal tumors with huge intrathoracic involvement: a surgical intervention for neurological decompression and oncological control

**DOI:** 10.3389/fneur.2025.1661864

**Published:** 2025-10-08

**Authors:** Fangzhi Liu, Ben Wang, Jiacheng Liu, Xiaoguang Liu, Fengliang Wu, Hua Zhou, Lei Dang, Yan Li, Yanchao Tang, Xiao Liu, Panpan Hu, Zihe Li, Feng Wei, Zhongjun Liu

**Affiliations:** ^1^Orthopedic Department, Peking University Third Hospital, Beijing, China; ^2^Engineering Research Center of Bone and Joint Precision Medicine, Peking University, Beijing, China; ^3^Beijing Key Laboratory of Spinal Disease Research, Peking University, Beijing, China

**Keywords:** primary spinal tumors, huge intrathoracic involvement, en bloc resection, spinal cord compression, neurological deficit, surgical approach, complication, recurrence

## Abstract

**Study design:**

Retrospective cross-sectional study.

**Objectives:**

Given the aggressive nature of primary spinal tumors, postsurgical local recurrence rates remain high. En bloc resection is currently the preferred treatment. However, the presence of a large thoracic cavity mass increases the surgical difficulty, risk, and likelihood of extensive complications. We report diagnostic and therapeutic characteristics, surgical strategies, and perioperative complications of such tumors treated with en bloc resection.

**Methods:**

We reviewed 25 patients with primary spinal tumors and extensive thoracic cavity involvement who underwent en bloc resection at our center between 2012 and 2023 with a minimum postoperative follow-up of 1 year. We collected and analyzed data on surgical procedures, complication characteristics, and local tumor control and recurrence, and compared our findings with previous studies.

**Results:**

We included 25 patients (14 males and 11 females; mean age, 41.3 years). Of these, 14 patients underwent the first surgery, and 11 experienced recurrences. All patients underwent en bloc resection; 9 and 16 underwent intralesional and extralesional resections, respectively, 16 and 9 underwent posterior-only and combined approaches, respectively. The average surgery duration was 674 min, with an average estimated intraoperative blood loss of 2,388 mL. Eighty complications were recorded; 24 patients (96%) experienced at least one perioperative complication.

**Conclusion:**

For primary spinal tumors with huge thoracic cavity involvement, en bloc resection remains the optimal treatment for achieving local tumor control. Suitability for this procedure depends on the patient’s fitness for major surgery, the absence of distant metastases, and tumor resectability. Surgery can be performed via posterior-only or combined anteroposterior approaches.

## Introduction

En bloc resection is the preferred treatment approach for primary spinal tumors (1, 2). This procedure is designed to excise the tumor entirely along with the affected vertebra and adjacent tissue lesions, providing adequate margins, thereby significantly reducing the risk of local tumor recurrence (3). However, en bloc resection entails substantial resection and trauma, making it a highly complex procedure with a significant risk of complications. Furthermore, the complexity of the operation varies considerably depending on the specific surgical site and segments involved.

Compared with the cervical and lumbar vertebrae, the proximity of the thoracic vertebrae to essential structures, such as the pleura, lungs, heart, major blood vessels, trachea, and lymphatic vessels, markedly increases the complexity and associated risks of surgical interventions. In particular, when thoracic spine tumors manifest as large intrathoracic masses, they may adhere to or breach the pleura and infiltrate the lung tissue. In such scenarios, irrespective of using a purely posterior or combined surgical approach, the risk is exceptionally high, and intraoperative pleural damage is unavoidable, potentially leading to damage to the intrathoracic blood vessels, nerves, and thoracic duct, culminating in severe postoperative complications, including pneumothorax, pleural effusion, neurological dysfunction, and chylothorax. The complication rate of en bloc spinal tumor resection ranges 46–87% (4–9). The unique anatomical location of intrathoracic tumors significantly increases the difficulty of nonsurgical tumor resection and the likelihood of local recurrence and also leads to a higher incidence of perioperative complications. Therefore, perioperative management presents a formidable challenge.

Primary spinal tumors are rare, and relatively few reports are published reports on primary spinal tumors with extensive intrathoracic involvement. Our center has a significant caseload of primary spinal tumors and boasts considerable expertise in their management. Through the analysis of diagnostic and therapeutic strategies for these cases, coupled with this retrospective study, we aimed to report the treatment protocols and results for primary spinal tumors with huge intrathoracic involvement.

## Materials and methods

### General information

This study was approved by the Ethics Committee of the University’s hospital (IRB00006761-M2023022) and its implementation adhered to the Declaration of Helsinki. The informed consent was waived because the retrospective nature of the study.

A review of the spinal tumor case data at our center, spanning from January 1, 2012, to August 31, 2023, revealed that our spinal surgery team performed en bloc resection on 140 cases of primary spinal tumors, including 31 cases of primary thoracic spine tumors. Patients treated prior to 2012 could not be followed up because of incomplete early electronic database records and changes in contact information. Ultimately, we identified 25 patients with spinal tumors with confirmed intrathoracic space occupation who underwent en bloc resection vertebrectomy and reconstruction in our department. The inclusion criteria were as follows: primary malignant and aggressive benign spinal tumors confirmed through final histological diagnosis, clear evidence of huge intrathoracic space occupation observed on imaging (here, we define huge intrathoracic involvement on the axial computed tomography (CT) images or magnetic resonance imaging (MRI) scans, the maximum diameter of the tumor was greater than or equal to the corresponding vertebral diameter) or during surgery, a postoperative follow-up period of at least 12 months, and sufficient radiological data. Patients with metastatic spinal tumors, tumors that did not involve the thoracic cavity, or those who underwent non-en bloc resection surgeries were excluded.

The retrospective evaluation included electronic medical, surgical, and anesthesia records; pathology reports; and radiological images. The collected data included patient age, gender, clinical presentation, underlying diseases, smoking habits, body mass index, neurological function, radiological characteristics, Enneking staging, surgical approach, duration of surgery, estimated intraoperative blood loss, postoperative pathology, time from surgery to discharge, and treatment complications. Neurological function was evaluated using a modified Frankel scoring system. Enneking staging was performed based on radiological findings.

### Imaging and biopsy

Radiography, CT, and MRI were routinely performed in all patients. When indicated, bone scans and positron-emission tomography CT scans are conducted to exclude systemic metastases. Computed tomography angiography is performed when the tumors are anatomically proximal to the major vascular structures. CT-guided percutaneous biopsy of the tumor tissue was performed in patients lacking a clear preoperative histopathological diagnosis.

### Treatment protocol

In clinical practice, for primary spinal tumors, an initial assessment of the patient’s clinical presentation was required. If the patient experiences an acute exacerbation of pain, sensory and motor disturbances, or a complete loss of neurological function, emergency surgery was performed. If the patient’s condition is stable and symptoms progress slowly, a CT-guided biopsy was routinely conducted to establish a definitive diagnosis and further delineate treatment and surgical plans. Surgical decision making occurred through a collaborative approach with a multidisciplinary team (MDT) (10) comprising spine, thoracic, and vascular surgeons; oncologists; radiation therapists; and anesthesiologists.

Indications for en bloc resection include primary malignant or aggressive benign tumors without metastasis. All the surgeries were performed by experienced surgeons. The surgical approach, including the choice of approach, was tailored based on the tumor’s location, size, extent of involvement, the patient’s baseline health status, and the results of MDT discussions, while referencing the Weinstein-Boriani-Biagini (WBB) staging system (3, 11). Routine preoperative embolization of the tumor-supplying arteries was performed to block the tumor blood supply, inhibit its growth, and reduce the risk of bleeding during surgery (12). Reconstruction of the spine after tumor resection involved either titanium meshes or 3D-printed artificial vertebral bodies (AVBs). In the earlier period of this study, off-the-shelf 3D-printed AVBs were utilized, while in the later period, patient-specific customized AVBs were designed and implanted based on preoperative CT imaging.

For patients with giant cell tumor of bone (GCT), preoperative denosumab was administered in selected cases to induce tumor ossification and reduce intraoperative vascularity. In our series, 4 of the 12 GCT patients received denosumab therapy. The duration of preoperative treatment ranged from 3 to 5 months.

### Surgical technique

For the posterior-only approach, patients were placed in the prone position. A midline incision was made, followed by subperiosteal dissection to expose the posterior elements of the spine, typically extending two levels above and below the lesion. Pedicle screw instrumentation was placed first to ensure spinal stability. A wide laminectomy of the involved vertebrae was performed to decompress the spinal cord and expose the tumor. The nerve roots at the tumor level were ligated and transected. Osteotomies of the pedicles and the posterior vertebral body wall were then performed. The intervertebral disks above and below the lesion were incised, and the anterior longitudinal ligament was released. The tumor-involved vertebral body was then carefully dissected from the pleura and major vessels and removed en bloc.

For combined anterior–posterior approaches, the procedure was typically staged. The anterior stage was performed first with the patient in the lateral decubitus position. A thoracotomy was performed to access the thoracic cavity. The segmental vessels supplying the tumor were identified and ligated. The intervertebral disks were resected, and the tumor was meticulously dissected and mobilized from the anterior vital structures, such as the aorta, vena cava, and esophagus. Following the anterior release, the patient was repositioned to the prone position for the posterior stage, which proceeded with laminectomy, osteotomies, and final en bloc removal of the specimen as described above. Spinal reconstruction for all approaches typically involved the use of a 3D-printed vertebral body or a titanium mesh cage, followed by posterior fixation with rods and screws.

Management of the chest wall defect was dependent on the size and location of the resection. For defects involving the removal of three or fewer posterior rib segments, which was the most common scenario in our series, formal reconstruction was typically not required. The overlying scapula and robust paraspinous musculature were deemed sufficient to provide adequate stability and prevent herniation of lung tissue. In cases where a larger anterolateral chest wall defect was created that posed a risk of paradoxical respiratory motion (flail chest), reconstruction was performed using a prosthetic mesh (e.g., polypropylene mesh) sutured to the rib margins to restore thoracic cage integrity. In all cases involving entry into the thoracic cavity, meticulous layered closure of the musculature was performed, and one or more thoracic drains were placed to manage pleural effusion and prevent pneumothorax.

All tumor classifications were determined by the final pathological diagnosis according to the WHO Classification of Tumors of Bone and Soft Tissue (2020) (13). According to McDonnell’s standards (14), complications were divided into major and minor categories depending on whether they significantly affected patient recovery.

### Follow-up

Imaging examinations were conducted at 3, 6, and 12 months postoperatively, and annually thereafter. MRI was performed at the 3-month follow-up, and subsequently annually. If a patient exhibited symptoms suggestive of local recurrence, MRI was performed immediately, with imaging characteristics of recurrence including the progressive increase in the size of the treated lesion, the appearance of new soft tissue masses, nodular enhancement, or changes in signal intensity on T2-weighted and contrast-enhanced T1-weighted sequences. The minimum follow-up duration was 12 months.

### Statistical analysis

All collected data were analyzed using SPSS Statistics (version 25.0; SPSS Inc., Chicago, IL, United States). The Kolmogorov– Smirnov test was used to determine if the data followed a normal distribution. For normally distributed data, quantitative variables were presented as means and standard deviations, whereas for data that were not normally distributed, they were presented as medians along with maximum or minimum values. Statistical significance was set at *p* < 0.05.

## Results

### Demographic and clinical characteristics

This study included 25 patients (14 male and 11 female participants) with a mean age of 41.3 years. Detailed demographic and clinical characteristics are summarized in Table 1. All patients demonstrated neurological deficits attributable to the occupation of the thoracic spinal tumor. Preoperatively, 13 patients displayed signs of spinal cord symptoms (Frankel grades A-D), while 12 patients were neurologically intact (Frankel grade E). Four and two patients exhibited symptoms related to cauda equina syndrome and nerve root damage, respectively. Thirteen patients also reported pain. The mean duration from symptom onset to surgery was 15.78 months (range 1–156 months). Fifteen patients underwent initial surgical treatment, while the remaining 10 patients underwent revision surgeries following previous operations in other hospitals.

**Table 1:** Demographic, clinical and surgical characteristics.

Items		Values
Gender (male: female)		14:11
Age (years), mean ± SD		41.3 ± 13.7
Postoperative hospital stay (days), median (IQR)		15(9)
Preoperative embolization, *n* (%)		15(60.0)
Surgical duration (mins), mean ± SD		674 ± 302
Estimated blood loss (mL), mean ± SD		2,388 ± 1,692
Preoperative neurological function, *n* (%)		
Frankel A		2(8.0)
Frankel B		2(8.0)
Frankel C		2(8.0)
Frankel D		7(28.0)
Frankel E		12(48.0)
Location of tumor, *n* (%)		
Thoracic spine	Single-segment	10 (40.0)
	Multi-segment	13(52.0)
Thoraco-lumbar spine (multi-segment)		2(8.0)
Surgical resection margin, *n* (%)		
Trans-tumoral		9(36.0)
Non-trans-tumoral		16(64.0)
Surgical approach, *n* (%)		
Posterior alone		16(64.0)
Combined approach		9(36.0)

The results are presented as the mean ± standard deviation, median (InterQuartile Range), number (percentage), or number only. Abbreviations: SD: standard deviation; IQR: InterQuartile Range.

Preoperative biopsy pathology results were available for 19 (76%) patients. The final pathological diagnoses for all 25 patients are detailed in Table 2 and included giant cell tumor (*n* = 12), chondrosarcoma (*n* = 3), chordoma (*n* = 2), malignant spindle cell sarcoma (*n* = 2), malignant hemangioendothelioma (*n* = 2), osteosarcoma (*n* = 1), osteoblastoma (*n* = 1), schwannoma (*n* = 1), and ameloblastoma (*n* = 1). All patients underwent en bloc resection via a posterior-only approach (*n* = 16) or a combined approach (*n* = 9). Owing to tumors adhering to the parietal pleura or penetrating the visceral pleura, 14 patients (56.0%) experienced intraoperative tearing or damage to the pleural layers, including one case of lung tissue damage, which was promptly repaired by a surgeon or thoracic surgeon. In five cases (20.8%), dural damage or nerve root sleeve tears occurred during tumor resection, leading to cerebrospinal fluid leaks, which were repaired intraoperatively, and the patients subsequently recovered with conservative postoperative treatment.

**Table 2:** Characteristics of all cases.

No.	Age	Gender	Tumor	Surgical way	Surgical time (min)	Blood loss (ml)	Major complication	Duration of chest drainage (d)	Postoperative hospital stay (d)	Follow-up	Outcome
1	35	M	Giant cell tumor	A&P	1,413	3,400	Atelectasis	7	17	115	Well
2	36	M	Giant cell tumor	P	1,263	4,400	/	12	27	47	Well
3	41	M	Chondrosarcoma	P	443	3,000	Pleural injury, Pleural effusion, Improper internal fixation	/	15	59	Well
4	55	M	Giant cell tumor	P	945	5,000	Major vascular injury, Postoperative bleeding, Improper internal fixation	8	28	36	Well
5	49	M	Giant cell tumor	P	750	2000	Improper internal fixation	6	12	56	Well
6	22	M	Giant cell tumor	P	365	3,000	Pleural injury, Pleural effusion	0	11	66	Well
7	55	F	Giant cell tumor	P	471	1,200	/	/	19	91	Well
8	42	M	Malignant hemangioendothelioma	P	338	1,000	Pleural injury	/	6	22	Well
9	49	M	Osteosarcoma	P	540	3,000	Pleural injury, Pleural effusion, Dural tear	11	25	67	Death
10	22	F	Giant cell tumor	L	711	1,100	Lung rupture	/	18	65	Well
11	71	F	Chordoma	A&P	685	800	Respiratory infection, Improper internal fixation	/	24	/	/
12	39	M	Malignant hemangioendothelioma	L&P	658	400	Pleural injury, Pleural effusion	12	26	50	Well
13	29	F	Giant cell tumor	P	392	1,000	/	7	12	34	Well
14	53	F	Chondrosarcoma	L&P	1,154	3,300	Pleural injury, Pleural effusion, Postoperative neurological dysfunction	13	14	2	Death
15	63	M	Chondrosarcoma	P	419	1,300	Postoperative neurological dysfunction, Deep venous thrombosis	5	6	18	Well
16	30	M	Giant cell tumor	P	757	3,600	Major vascular injury	6	11	26	Well
17	36	M	Malignant spindle cell sarcoma	P	1,100	5,500	Major vascular injury, Postoperative neurological dysfunction	35	87	12	Death
18	15	F	Giant cell tumor	P	446	500	Pleural injury, Pleural effusion	4	8	8	Well
19	36	M	Giant cell tumor	P	275	2000	Pleural injury, Pleural effusion, Postoperative neurological dysfunction	/	6	17	Well
20	40	F	Malignant spindle cell sarcoma	A&P	689	1,000	Cardiac arrest	4	13	0	Death
21	24	F	Osteoblastoma	L&P	636	1,100	Pleural injury, Pleural effusion	8	14	12	Well
22	56	F	Schwannoma	P	367	1,000	Pleural injury, Pleural effusion, Atelectasis	4	10	12	Well
23	54	M	Chordoma	A&P	945	7,000	Pleural injury, Pleural effusion, Major vascular injury, Deep venous thrombosis	12	20	12	Well
24	30	F	Ameloblastoma	P	510	1,300	Pleural injury, Pleural effusion	/	15	12	Well
25	50	F	Giant cell tumor	A&P	586	2,800	Pleural injury, Pleural effusion	7	15	49	Well

The results are presented as number only. The unit of follow-up time is months. No., number; M, male; F, female; A, anterior approach; P, posterior approach.

### Perioperative complications

**Figure 1:**
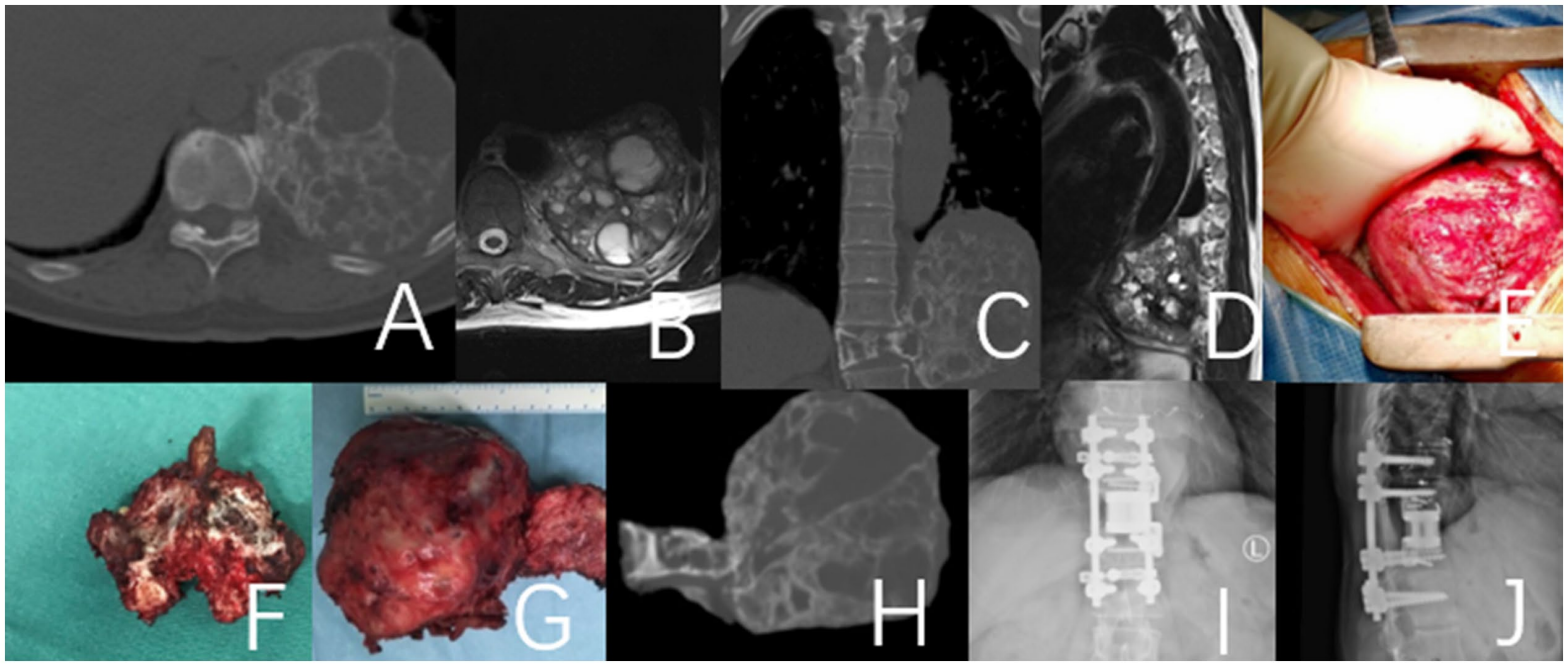
This case involves a 50-year-old female patient with a T11 giant cell tumor, classified under the WBB staging system as T11 1–10 **(A–D)**. The patient received denosumab treatment for 3 months prior to undergoing staged T11 en bloc resection surgery, during which a 3D-printed artificial vertebra was implanted between T10 and T12, and subsequently fixed. During the surgery, tumor operations were executed while cutting both sides of the pedicles. During posterior approach surgery, a tear in the right pleura resulted in pleural effusion, which was resolved with adequate drainage, leading to a full recovery. **(A–D)** Preoperative CT and MRI images revealed a large mass in the thoracic cavity formed by the T11 vertebral tumor. **(E)** Second-phase anterolateral approach surgery. **(G)** Specimen of the second phase surgery. **(F)** Specimen of the first phase posterior surgery. **(H)** CT images of the T11 vertebra and tumor excised during the second-stage surgery. **(I,J)** Imaging at the 4-year postoperative follow-up indicated that the internal fixation was well positioned and showed no evidence of tumor recurrence.

**Figure 2:**
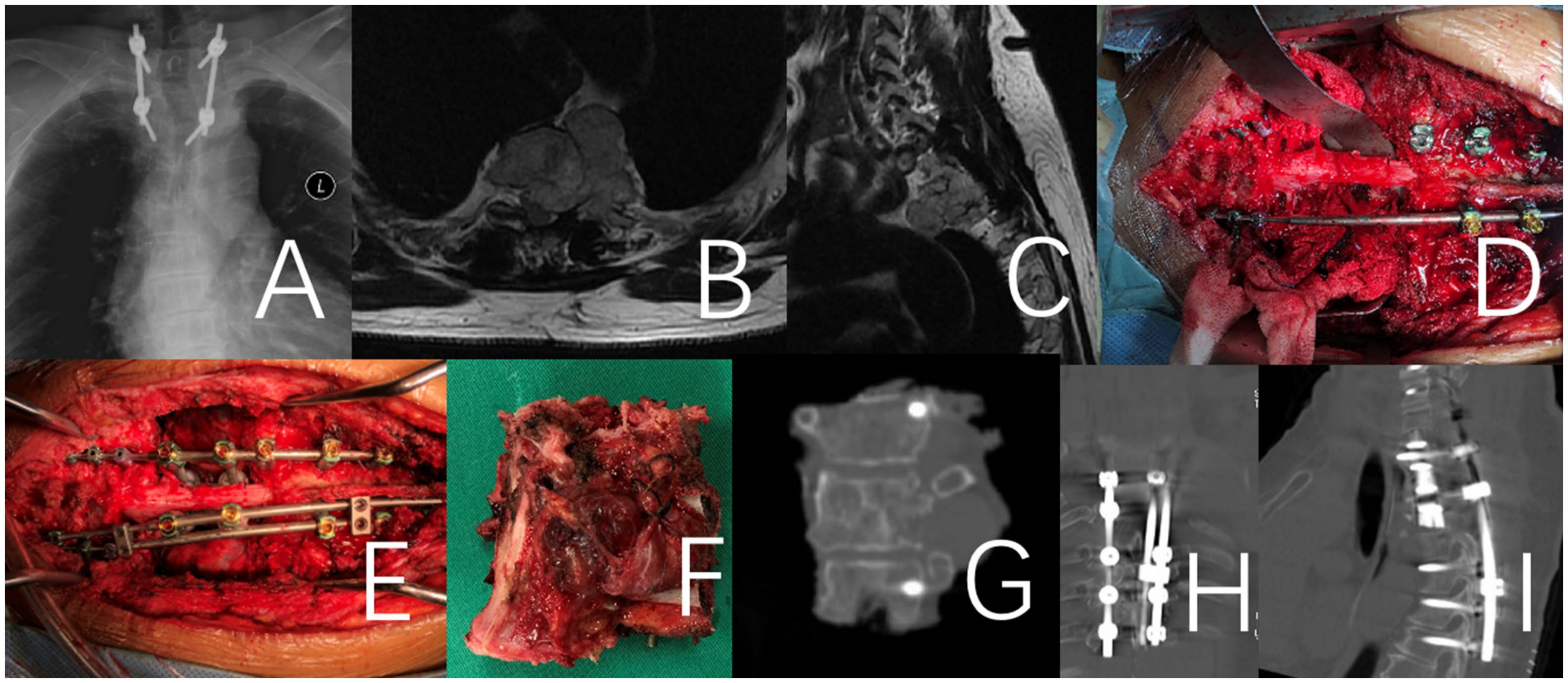
This case involves a 71-year-old female patient with a large T2 chordoma, classified as WBB stage T2 5–12 **(A–E)**. The patient underwent a one-stage en bloc resection. It is a revision surgery. Initially, a T1-3 tumor resection was performed via an anterior approach. Following anesthesia preparation, the patient was placed in a supine position, and a midline anterior cervical incision was made to fully expose the affected vertebrae and adjacent segments. In the second stage of the procedure, the patient was positioned prone for a posterior approach. The T1-3 vertebrae and the tumor were removed en bloc, and a 3D-printed artificial vertebra was implanted and fixed between C7 and T4. Throughout the procedure, there was no direct intralesional operation of the tumor. **(A–C)** Preoperative X-Ray and MRI. **(D–F)** Intraoperative imaging of the specimen after removal and an anterior view of the specimen. **(G)** X-ray image of the surgical excision specimen. **(H,I)** Illustrate the internal fixation after revision surgery.

**Figure 3:**
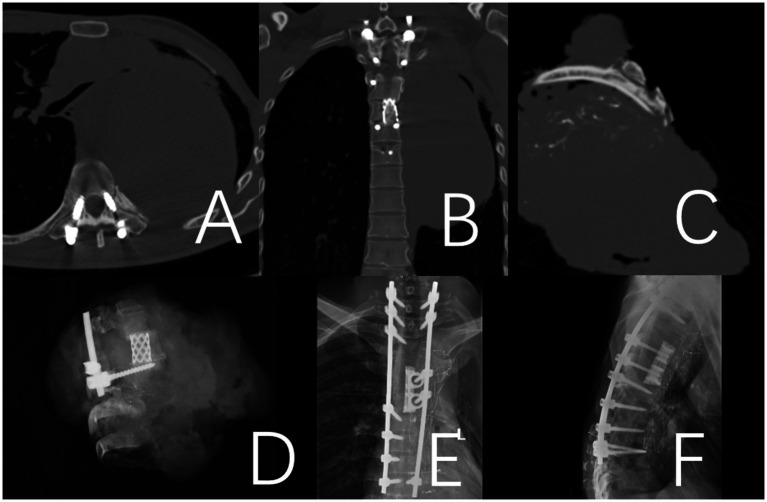
A 36-year-old male patient who presented with a recurrence 12 months after the initial surgery for a T2-10 spindle cell sarcoma. To address the recurrent lesion, which was primarily located at the T4-9 levels, a posterior approach T4-9 en bloc resection (partial through-tumor) and pulmonary metastasectomy were performed. A and B: Illustrate the internal fixation after revision surgery. **(A,B)** Preoperative CT revealed a paraspinal space-occupying lesion on the left side of the spine, which was considered malignant. **(C)** CT image of the surgical excision specimen. **(D)** X-ray image of the surgical excision specimen. **(E,F)** Illustrate the internal fixation after revision surgery.

A total of 80 complications were recorded (Table 3), with a mean of 3.2 perioperative complications per patient. These included 47 major and 33 minor complications. Nearly all (24) patients experienced at least one perioperative complication, with 3, 7, and 14 patients encountering one, two, and three or more complications, respectively. Major complications were observed in 20 patients (80.0%). One perioperative death occurred due to complications (Figures 1–3).

**Table 3:** Perioperative complications.

Perioperative complications		*n* (%)	Treatment	Outcome
Major complications				
Pleural injury		13(52.0)	All suture repairs	
Major vascular injury		4(16.0)	All vascular suture	All recovered
Respiratory system complications	Respiratory infection	1(4.0)	Anti-infection and oxygen inhalation	Recovered
	Lung rupture	1(4.0)	All suture repairs	Recovered
	Atelectasis	2(8.0)	Oxygen inhalation and lung expansion therapy	All recovered
Improper internal fixation		4(16.0)	Surgical adjustment	All recovered
Pleural effusion with pleural injury		13(52.0)	Puncture drainage or closed thoracic drainage	All recovered
Postoperative bleeding		1(4.0)	Discontinuation of anticoagulant medication, Conservative treatment	Recovered
Deep venous thrombosis		2(8.0)	Immobilization and anticoagulation	1 improved; 1 died due to PTE
Cardiovascular system complications (cardiac arrest)		1(4.0)	Cardiopulmonary resuscitation	Died
Postoperative neurological dysfunction	Trunk muscle weakness	3(12.0)	Neurological rehabilitation therapy	2 improved; 1 unchanged before discharge
	Respiratory muscle weakness	1(4.0)	Oxygen inhalation and neurological rehabilitation therapy	Recovered
Minor complications				
Cerebrospinal fluid leakage		6(24.0)	Dural sutures and drainage	All recovered
Dural tear		4(16.0)	All suture repairs	All recovered
Simple pleural effusion		9(36.0)	Conservative treatment	All recovered
Nutrition-related complications	Anemia	3(12.0)	Blood transfusions	All recovered
	Hyponatremia	2(8.0)	Sodium supplementation	All recovered
	Hypokalemia	1(4.0)	Potassium supplementation	Recovered
	Hypoproteinemia	4(16.0)	Albumin infusion	All recovered but 1 died after cardiac arrest
Chylous leakage		1(4.0)	Long-term indwelling drainage, nutritional support, diet control and other treatments	Recovered
Wound healing problems		2(8.0)	Debridement	Recovered
Urinary tract infection		2(8.0)	Anti-infection treatment	1 recovered but 1 died after cardiac arrest

The results are presented as number (percentage).

The most frequent complications were thoracic-related. Vascular injuries occurred in four cases (16.0%), leading to significantly higher blood loss (*p* = 0.02) and longer surgical duration (*p* = 0.026) in these patients compared to others. Neurological function deteriorated postoperatively in four patients (16.0%). Other significant complications included deep vein thrombosis (*n* = 2, 8.0%), which led to a fatal pulmonary embolism in one patient, and poor wound healing requiring debridement (*n* = 2, 8.0%). Detailed information on all perioperative complications and their management is presented in Table 3.

### Follow-up and late complications

One patient succumbed to pulmonary embolism 13 d postoperatively, another to tumor recurrence 2 months postoperatively, and a third was followed up for 9 months, showing no signs of recurrence at the last follow-up. One patient was lost to follow-up due to failure to return for examination and subsequent inability to make contact. The remaining patients were followed-up for a minimum of 12 months, with an average follow-up period of 38.6 months.

**Figure 4:**
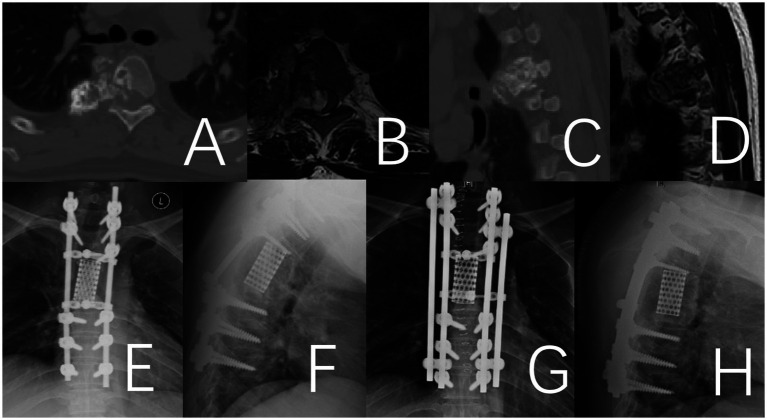
Fracture of the titanium rod observed 38 months after the operation in a 41-year-old male patient with thoracic chondrosarcoma. **(A–D)** Preoperative CT and MRI. **(E,F)** Depict the fractured titanium rod. **(G,H)** Illustrate the internal fixation after revision surgery.

**Figure 5:**
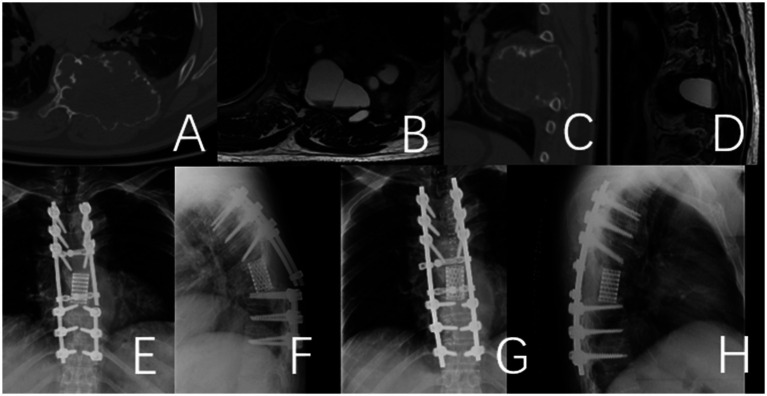
Fracture of the titanium rod observed 26 months after the operation in a 49-year-old male patient with thoracic giant cell tumor. **(A–D)** Preoperative CT and MRI. **(E,F)** Depict the fractured titanium rod. **(G,H)** Illustrate the internal fixation after revision surgery.

Two patients experienced hardware failures within 26 and 38 months postoperatively, characterized by fractures in the titanium fixation rods, which necessitated revision surgery (Figures 4, 5). Both patients who experienced hardware failure had undergone initial reconstruction with titanium mesh. In contrast, no fixation failure was observed in patients who received a 3D-printed vertebral body for reconstruction.

### Local tumor control

**Figure 6:**
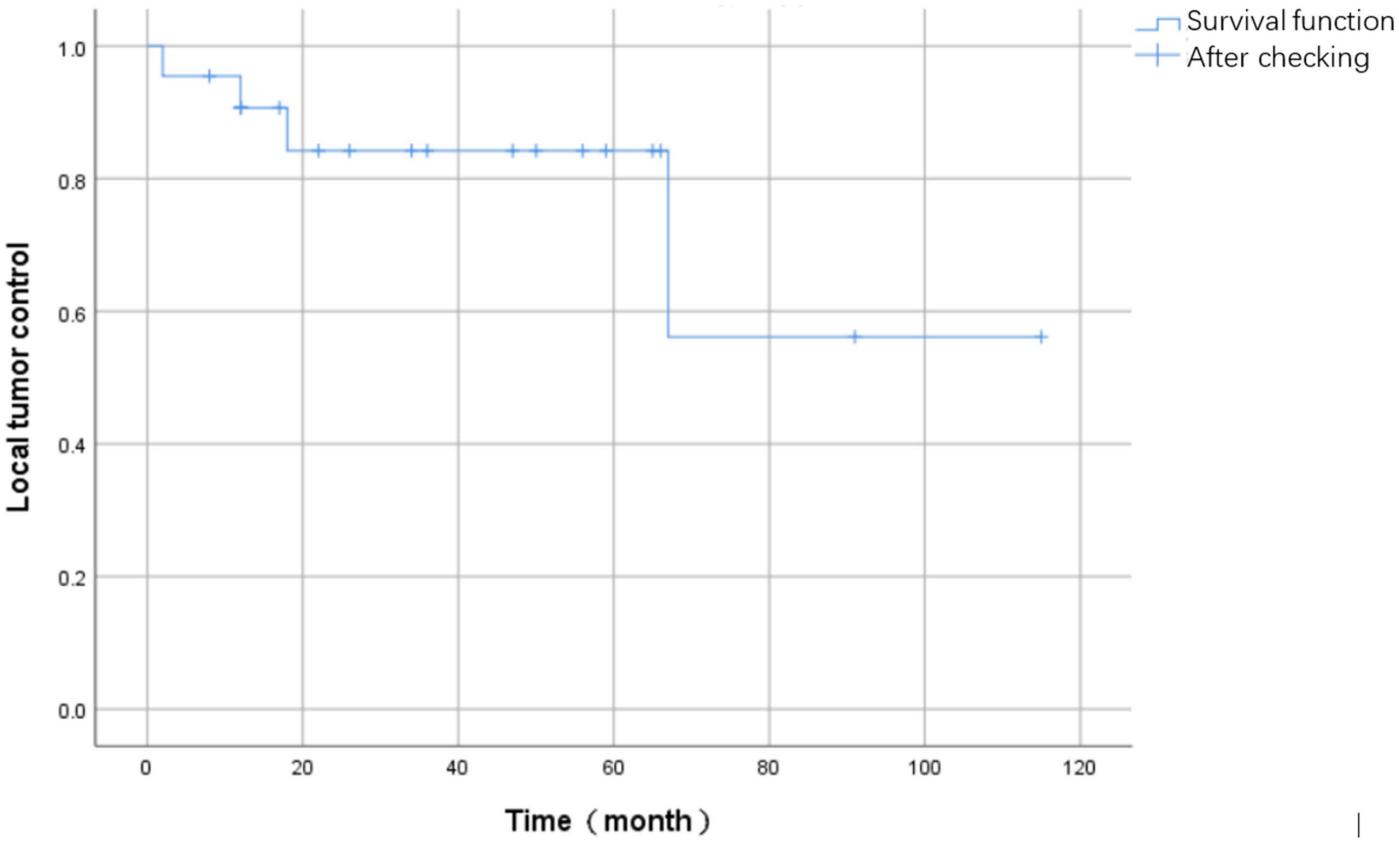
The Kaplan–Meier curve depicting survival events associated with tumor recurrence.

During the follow-up period, four patients experienced tumor recurrence, resulting in a recurrence rate of 16.0% (4/25). The mean time to recurrence was 24.8 months (SD = 25.1) and the median time was 15 months (IQR = 20.8). The Kaplan–Meier curve depicting survival events associated with tumor recurrence is presented in Figure 6.

Regarding the four patients who experienced local recurrence, the specific time points and MRI findings were as follows: One patient experienced recurrence at 2 months postoperatively; MRI revealed an irregular soft tissue mass within and adjacent to the T2-4 vertebral bodies. The mass exhibited heterogeneous signal intensity, being predominantly hypointense on T1-weighted and hyperintense on T2-weighted images, with heterogeneous enhancement on contrast-enhanced scans. Another patient had a recurrence at 12 months, with MRI demonstrating a large, heterogeneous soft tissue mass in the left paravertebral region, which partially extended into the spinal canal. The third case of recurrence occurred at 18 months postoperatively, where MRI identified multiple nodules with hypointense T1 and hyperintense T2 signals in the right posterior aspect of the T3-5 posterior elements. The fourth patient developed a recurrence at 67 months, with MRI showing bony destruction of the T7 and T9 vertebral bodies, the right posterior elements, and the posterior aspect of the 8th right rib. An associated irregular soft tissue mass was observed locally and in the right paravertebral region, displaying hypointensity on T1-weighted and slight hyperintensity on T2-weighted images, with the mass encasing the spinal canal.

Three patients with local recurrences underwent radiotherapy and/or chemotherapy. By the last follow-up, all three patients had succumbed to the disease, which was attributed to local tumor recurrence and deterioration of their general condition.

Another patient achieved satisfactory local control of the tumor but subsequently developed distant metastases. A 49-year-old male with thoracic osteosarcoma was diagnosed with abdominal wall metastasis 1 month after en bloc resection. Despite expanded resection of the abdominal metastasis along with chemotherapy and radiotherapy, the patient ultimately succumbed to thoracic osteosarcoma metastasis 66 months postoperatively.

The clinical characteristics, surgical interventions, and prognostic outcomes of all patients are summarized in Table 2.

To analyze the impact of pathological type on prognosis, patients were stratified into two groups according to the WHO Classification of Tumours of Bone: malignant tumors and intermediate (locally aggressive) tumors. The malignant group (*n* = 10) included patients with osteosarcoma, chondrosarcoma, chordoma, malignant spindle cell sarcoma, and malignant hemangioendothelioma. The intermediate group (*n* = 15) comprised patients with giant cell tumor of bone, osteoblastoma, schwannoma, and ameloblastoma. A comparative analysis was performed to assess differences in surgical duration, intraoperative blood loss, number of perioperative complications, postoperative recurrence, and long-term survival between the two groups.

The results indicated no statistically significant differences in surgical difficulty or the number of complications. However, the malignant tumor group exhibited a significantly higher proportion of postoperative recurrence compared to the intermediate tumor group (5/10 vs. 0/15, *p* = 0.002). Similarly, the mortality rate at the final follow-up was also significantly higher in the malignant group (5/10 vs. 0/15, *p* = 0.002).

### Illustrative cases of surgical approach selection

The choice of surgical approach was tailored to the specific anatomical challenges presented by the tumor, particularly for the nine cases requiring combined approaches. For example, in patient 12, a primary tumor at T3–T4 was located in close proximity to the aortic arch. A sole posterior approach was deemed insufficient for complete tumor separation. Consequently, a left anterolateral approach was first employed for tumor dissection and vascular control, followed by a posterior approach for the en bloc spondylectomy.

Similarly, patient 14, who had a recurrent chondrosarcoma from T2 to T4 with extensive invasion into the pleura and left lung, required a two-stage transthoracic-posterior combined approach. The initial transthoracic stage allowed for mobilization and partial resection of the invaded lung tissue, while the second posterior stage facilitated the vertebral resection.

Finally, in patient 21, a recurrent osteoblastoma spanning T5–T7 was excised using a combined approach. These cases highlight the necessity of combined or staged approaches to achieve safe and complete resection for tumors with complex anterior and lateral extension.

## Discussion

Although the incidence of primary thoracic spine tumors is relatively low, their surgical treatment is challenging, often resulting in poor prognosis, high mortality rate, and significant disease burden. Surgical resection includes three principal techniques: En bloc resection, sagittal resection, and posterior arch resection. Notably, the en bloc resection technique is distinguished by its extensive resection margins, capable of entirely removing the tumor in a single-stage operation, although complex tumors may necessitate a two-stage procedure (2, 3, 15). The posterior approach, with the patient in the prone position, entails the removal of the posterior spinal structures, including the annulus fibrosus and posterior longitudinal ligament, facilitating effective hemostasis of the epidural venous plexus and spinal stabilization through posterior reconstruction (16–20). The anterior approach, via thoracotomy and retroperitoneal or thoracoabdominal access, facilitates ligation of the vessels of the affected vertebrae and adjacent segments, resection of the proximal and distal intervertebral disks, and ultimately, complete resection and anterior reconstruction of the vertebral body. The combined anterior–posterior approach is especially effective in ligating segmental vessels and separating the tumor from the anterior aspect under direct vision, crucial for achieving wide negative margins when the tumor extends anteriorly (7, 21, 22). We systematically reviewed and summarized the existing literature (23–32) (Table 4). However, most existing studies are single-case reports with limited sample sizes. Consequently, specific surgical resection methods, the choice of approach, treatment outcomes, postoperative results, and their relationship with complications have not yet been substantiated in large-scale studies.

**Table 4:** Summary of reported cases of primary tumor of thoracic spine.

Authors	Year	Age	Gender	Tumor and location	Treatment and surgical way	Resection	Complication	Follow-up	End
Kumar et al. (32)	2007	16	F	T4-5 Primary osseous hemangiopericytoma	T3-5 laminectomy P	Complete	None	9	Well
Ali et al. (29)	2009	31	M	Thoracic spine primary melanoma	Surgery No record	Incomplete	Recurrence	1	Death
Huang et al. (34)	2011	24	M	T6-7 Primary melanoma	T6–T7 laminectomy No record	Incomplete	None	0.5	Well
Ahmadi et al. (24)	2012	65	M	T3-7 Marginal zone lymphoma	T4-6 laminectomy, radiation therapy No record	Incomplete	None	84	Well
Uehara et al. (31)	2013	28	F	T1-3 Hodgkin’s disease	Spinal cord decompression therapy A&P	Incomplete	None	120	Well
Halevi et al. (25)	2015	70	M	T4-6 Liposarcoma	T4–T5 laminectomy, T5–T6 foraminotomy No record	Incomplete	Recurrence	12	Death
Wang et al. (28)	2016	60	M	T1, T3-4 Primary melanoma	T1–T4 laminectomy No record	Complete	None	19	Well
Onoki et al. (23)	2018	25	M	T5-7 Primary osseous hemangiopericytoma	T5-7 two-stage TES A&P	Complete	None	24	Well
Liu et al. (26)	2023	19	M	T2-3 Osteosarcoma	T2-3 TES P	Complete	None	1	Well

The results are presented as number only. The unit of follow-up time is months. M, male; F, female; A, anterior approach; P, posterior approach.

When managing large and extensive primary thoracic spine tumors, single anterior or posterior surgical approaches frequently fail to achieve complete tumor resection because of the inevitable disruption of the tumor capsule. Therefore, the selection of an appropriate surgical approach is crucial for the successful execution of en bloc resection, ensuring the protection of vital paraspinal and intrathoracic vessels, nerves, and cardiopulmonary organs. En bloc resection techniques typically employ either a single posterior approach or a combined anteroposterior approach. Yang et al. (21) performed an additional lateral incision on the affected side during surgery in seven patients, confirmed by preoperative MRI or CT scans, to exhibit rib or pleural tumor invasion. By excising the normal tissue to expose the ribs, they effectively and feasibly conducted en bloc resection on the vertebral and chest wall invasion areas. Shah et al. (7) described a two-stage en bloc resection technique employing a wire saw for both posterior and anterior approaches, which was effective in 33 follow-ups, rendering the anterior spinal vessels clearly visible and preventing spinal cord damage. Zaidi et al. (22) analyzed the outcomes of two-stage posterior–anterior lateral combined en bloc resection versus single-stage surgery and achieved complete spinal fixation in seven patients. No orthotic failures occurred. At the final follow-up, there were no local disease recurrences or deterioration of neurological function, with outcomes comparable to those of posterior-only approaches. Xu et al. (19) introduced a surgical technique for total resection of massive paraspinal chondrosarcoma, employing a combined posterior median and Wiltse approach in 15 cases, ultimately achieving excellent local control.

A specific consideration in our cohort was the use of preoperative denosumab for patients with giant cell tumors. Denosumab is known to induce a sclerotic rim and extensive new bone formation within the tumor, which can potentially increase the difficulty of the osteotomies and dissection. However, in our experience, this effect was clinically advantageous. The induced ossification of the tumor capsule created a firmer, more defined margin, which we found facilitated a safer en bloc resection and significantly reduced intraoperative blood loss by decreasing the tumor’s vascularity. We did not observe a notable increase in surgical duration or perioperative complications in the subgroup of patients treated with denosumab. Therefore, while surgeons must be prepared for the harder consistency of the bone, we believe the benefits of neoadjuvant denosumab in creating a clear dissection plane and controlling hemorrhage outweigh the technical challenges in these highly vascular tumors.

The incidence of complications in spinal tumor surgery is notably high, with reported rates ranging 13.6–46.2% (33). A study by Boriani et al. (5) indicated that the major complication rate after total vertebral resection was 32.9% (71/216). In our study, thoracic complications were the most prevalent, constituting 84% (21/25) of cases, including pleural effusion, pleural adhesion, atelectasis, pulmonary infection, chylothorax, and respiratory dysfunction.

Pleural effusion is a prominent complication associated with thoracic spine tumor surgery. During thoracotomy procedures involving the resection of pleural or chest wall tissue to ensure complete tumor removal, the pleural cavity may become directly exposed to the surgical field, thereby increasing the likelihood of pleural effusion. Under these conditions, prophylactic chest drainage is essential. In this study cohort, patients who presented with severe pleural damage and a high risk of pleural effusion received a chest drain at the conclusion of the surgical procedure, which resulted in favorable outcomes. Compared with the overall study population, the 22 patients who developed pleural effusion maintained effective control over complications, including pleural fluid accumulation, without demonstrating a significant increase in the average length of the postoperative hospital stay. Of these patients, 20 were successfully discharged within 30 days postoperatively.

Surgical adhesions between the tumor and pleura or lungs frequently result in unavoidable tissue damage; however, preoperative arterial embolization and transthoracic approaches can mitigate bleeding and enhance surgical visibility, thereby reducing the risk of thoracic-related complications.

Neurological complications occurred in 12% (3/25) of the cases, where achieving an optimal surgical view and tumor-free margins sometimes necessitated stretching or even removal of the corresponding nerve roots, leading to postoperative neurological dysfunction. Additionally, six patients experienced dural tears during the separation of the tumor adherent to the dura mater, resulting in cerebrospinal fluid leakage. One case involved a patient with pleural effusion, hypoalbuminemia, and urinary tract infection who abruptly suffered respiratory and cardiac arrest postoperatively in the ward and ultimately succumbed. Additional complications included lower limb deep vein thrombosis and hardware fixation failure.

Finally, the complex nature of primary thoracic spine tumors, particularly when combined with intrathoracic space-occupying lesions, necessitates close collaboration among MDTs. Initial diagnoses and treatment plans are developed through discussions among the MDT, which includes the radiology, pathology, radiotherapy, and chemotherapy departments. During surgical treatment, the department of anesthesiology plays a crucial role in managing patient care during preoperative preparation, intraoperative maintenance, and postoperative recovery. Preoperative embolization of the tumor-supplying arteries and intraoperative major arterial repair require vascular surgery. At our center, preoperative arterial embolization was performed in 15 of the 25 patients with primary thoracic spine tumors. Furthermore, thoracic spinal tumors involving the pleura and lungs, especially those requiring transthoracic approaches, require expertise in thoracic surgery. The Critical Care Department provides essential support throughout the perioperative period. Efficient MDT surgery for spinal tumors can be achieved by integrating these specialties within a patient-centered framework, thereby enhancing and optimizing patient prognosis.

### Limitations

This was a single-center retrospective study, potentially introducing bias in the interpretation of the scientific findings. Future multicenter, large-sample prospective studies are required to validate these conclusions.

## Conclusion

En bloc resection is essential for minimizing local recurrence and preventing distant metastasis. Surgical treatment of large intrathoracic tumors is highly challenging, necessitating multiple approaches and exhibiting a high rate of complications. This study highlights the considerable advantages of employing lateral, anterior, and combined anterior–posterior approaches for tumors with extensive thoracic occupancy. Collaboration among MDTs is vital for navigating the increased risk of complications and effectively managing complex surgical demands.

## Data Availability

The raw data supporting the conclusions of this article will be made available by the authors, without undue reservation.
